# Requirements and Use Cases for eHealth Solutions in Flexible Assertive Community Treatment Teams: Design Science Study

**DOI:** 10.2196/77354

**Published:** 2026-01-26

**Authors:** Erlend Bønes, Conceição Granja, Terje Solvoll

**Affiliations:** 1 Norwegian Centre for E-health Research, University Hospital of North Norway, P.O. Box 35, Tromsø, N-9038, Norway, 47 97655680; 2 Faculty of Nursing and Health Sciences, Nord University, Bodø, Norway

**Keywords:** mental health, FACT team, eHealth, requirements specification, use cases, electronic health records, electronic whiteboards, design science

## Abstract

**Background:**

Health care delivery is often fragmented, with different services being delivered by different organizations. Various forms of teamwork are often used in health care, aiming to mitigate the challenges related to this fragmentation. One example of teamwork in mental health is Flexible Assertive Community Treatment (FACT). FACT is a model for comprehensive and integrated care for patients with long-term, serious mental illness. FACT teams deliver services using assertive outreach to treat patients who can be hard to reach by health care services. However, Norwegian FACT teams have issues with the current eHealth solutions related to the fragmentation of health care.

**Objective:**

This study aimed to identify requirements and develop use cases and use case diagrams for eHealth solutions that support effective teamwork within FACT teams, using them in a case study for collaborative health care delivery.

**Methods:**

A design science framework was used to explicate the problems of eHealth solutions in FACT teams. This included performing the subactivities of defining the problem precisely, positioning and justifying the problem, and finding root causes. Based on this explication, we derived a set of requirements, use cases, and use case diagrams for FACT teams.

**Results:**

We present the explication of the problems of eHealth in Norwegian FACT teams. Building on the results, we present functional and nonfunctional requirements for electronic health records, electronic whiteboards, video conference solutions, and digital questionnaires. Improved integration across these systems was identified as a recurring need. We also provide use cases and diagrams illustrating system use in practice.

**Conclusions:**

FACT teams in Norway require more integrated and tailored eHealth solutions. The requirements and use cases presented in this study offer a foundation for developing tools that better support the collaborative and mobile nature of FACT team operations.

## Introduction

Recent trends in health care emphasize integrated care [1] and patient-centered care [2]. Integrated care delivers health care services coordinated across various providers, while patient-centered care focuses on patient involvement and establishing strong relationships between the patient and health care workers [1,2]. Both approaches depend on effective teamwork, which is increasingly regarded as essential for delivering high-quality health services [1-3]. However, studies have revealed variation in how teamwork is interpreted and practiced in health care, encompassing aspects such as interaction, roles, and team fluidity [4].

The Flexible Assertive Community Treatment (FACT) model is a comprehensive and integrated approach to care provision for patients with long-term, serious mental illness, which relies on teamwork [5]. Most FACT teams target adults, although FACT youth teams target youth aged 12 to 25 years, demonstrating the model’s adaptability and effectiveness [6]. FACT teams deliver a wide range of services, including mental health care, using assertive outreach and targeting patients who are often hard to reach for traditional health care services. The care provided to patients of FACT teams varies according to their needs. In stable phases, patients receive individual case management from 1 team member. In phases when they need intensive follow-up, a collaborative approach is adopted, involving multiple team members in a shared caseload [5]. In Norway, FACT teams are managed within the public health care system and serve as a cooperation between primary care, managed by the municipalities, and specialist care, under the responsibility of the Regional Health Authorities. Norwegian FACT teams therefore consist of employees from both sectors, working together in an integrated team. In this paper, we focus on Norwegian FACT teams, hereafter referred to as FACT teams, as a case study of teamwork, owing to the well-defined structure and roles of these teams [7]. In addition to team organization, the delivery of care in Norway is shaped by standardized patient pathways for specific diagnoses. These pathways structure treatment through requirements for patient communication, clearly defined responsibilities, and set timeframes for service delivery. They aim to reduce treatment variation, strengthen user involvement, and improve coordination among services. Although no pathway is tailored specifically for FACT, patients in FACT teams follow general mental health pathways, which influence how their care is organized [8].

In a previous study [9], we described how FACT teams use a range of eHealth solutions, including electronic health record (EHR) systems [10], calendars [11], video conferencing solutions [12], digital questionnaires [13], and electronic whiteboards [14]. However, the effectiveness of these tools is limited by the fragmentation of health care, where services are delivered by different organizations at varying levels of care [15]. This fragmentation contributes to medical errors and poses particular risks for mental health patients, who often depend on multiple health and social care providers [16]. One consequence is that health care workers often have limited access to relevant patient data across systems [17]. Because many eHealth solutions are developed as stand-alone products from single vendors, they cannot always exchange data, thereby restricting the flow of information [17-20]. FACT teams are particularly vulnerable to these challenges, since they operate across both municipal and specialist care and rely heavily on mobile and outreach-based work. This increases the need for digital solutions that can support coordination and information sharing across organizational boundaries [9]. Addressing these issues highlights the challenge of integration, which we define as the connection of data, applications, and application programming interfaces (APIs) to improve system interoperability [15]. Thus, the goal of integration is to enable seamless data exchange between health care applications. Two examples of projects that address integration challenges in health care include a comprehensive set of requirements for data integration based on a systematic literature review [21] and the Valkyrie project, which aims to develop a technical prototype of an information and communications technology (ICT) architecture to improve health care delivery [22].

The development of eHealth solutions typically involves the specification of requirements [23] and use cases [24]. In this study, we define requirements as statements describing the functions or qualities that an eHealth system must fulfill to support FACT team operations. We distinguish between functional requirements, which specify what the system should do, and nonfunctional requirements, which describe how the system should perform. We define a use case as a description of how users interact with a system to achieve a specific goal, often illustrated through use case diagrams [24]. These concepts form the basis for this study.

This study aimed to define the requirements and use cases for eHealth solutions that support teamwork in health care settings, with the goal of improving data access for health care professionals and reducing fragmentation through integration. To achieve this, we applied the initial 3 steps of the design science framework, using teamwork as a case study, focusing on FACT teams. The design science framework is a stepwise approach to developing practical solutions to real-world problems, and it is particularly suited for eHealth research because of its systematic and rigorous structure. In this paper, we present the results of our investigation as a set of requirements and use cases that specify the proposed eHealth solutions. Our results aim to inform policymakers, with the intention of demonstrating the needs of FACT teams and thereby laying the foundation for the technical development of tailored eHealth solutions.

## Methods

### Design Science Framework

For this study, we used the design science framework [23] to guide the development of requirements and use cases for eHealth solutions for FACT teams. This framework was chosen as it aligns with our goal of creating user-centered eHealth solutions that are well adjusted to teamwork. It supports a structured approach and emphasizes an iterative process in artifact development. Design science consists of a set of iterative steps: (1) explicate the problem, (2) define requirements, (3) design and develop an artifact, (4) demonstrate the artifact, (5) evaluate the artifact, and (6) communicate artifact knowledge [23]. We focused on the initial 3 steps of this framework to inform the development of eHealth solutions that support teamwork in health care settings.

### Problem Explication

In the first step of the design science framework, we defined the problem that the eHealth solutions aim to address. We defined the problem precisely to make it clearer and easier to communicate; positioned the problem in a context; and justified the identified problem to demonstrate its significance, relevance, solvability, and level of challenge. The explication also included an analysis of its root causes, which provides additional context, ensuring focus on the causes of the problem rather than its symptoms. Unless otherwise specified, analytical steps were conducted collaboratively, with the first author leading the analysis and the coauthors contributing through iterative discussion and review.

To define the problem precisely, we built on the results of a previous thematic analysis of observations and interviews in both urban and rural FACT teams [9]. In that study, initial themes were defined and coded. During review, subthemes were added to differentiate distinct aspects of the data. In this study, these themes and subthemes formed the foundation for problem definition. The narratives underlying each theme and subtheme were analyzed in light of the existing eHealth infrastructure and regulations governing eHealth in Norway [19].

The initial identification of problem situations was conducted by the first author, who reviewed each theme and its associated coded extracts to identify instances where current digital tools hindered teamwork, coordination, or patient care. These preliminary interpretations were then presented to the coauthors, who provided methodological and domain-related feedback. Through iterative discussion in project meetings, the problem descriptions were refined as necessary. This process ensured analytic rigor by combining the detailed perspective of the primary analyst with validation from experienced researchers.

The resulting set of problem descriptions was subsequently grouped into broader categories representing the general shortcomings of current eHealth solutions. Table 1 provides an overview of the themes and subthemes applied in this study, and full details of their derivation have been reported previously [9].

**Table 1.** Themes and subthemes used for problem definition [9].

Main theme	Subthemes
Communication	Suitability of video consultations
Documentation	Documentation while traveling
Organization	Systems not adapted to FACT^a^ teams
COVID-19	Maintaining team capacity; maintaining contact with patients
Technologies	Electronic health records; electronic whiteboards; calendars; lack of integration

a FACT: Flexible Assertive Community Treatment.

Although no patient data were used in this study, ethical considerations regarding patient privacy and data security were considered when analyzing FACT team needs and designing requirements. Ensuring that eHealth solutions enable data sharing while protecting sensitive health information informed the extraction of problem descriptions and requirements.

To position the problem, we performed a document analysis [25] of 2 central sources: the FACT model description [5] and a national evaluation report of FACT teams [26]. The analysis followed a structured process of identifying, selecting, appraising, and synthesizing information from these documents. The documents were first read superficially to gain an overview and then examined in depth to extract descriptions of the purpose of FACT teams, their core activities, their organizational structure, stakeholder involvement, and the environments in which they operate. This contextualization ensured that the problem definition was not limited to the technical shortcomings of eHealth solutions but was situated in the organizational and operational realities of FACT teams, thereby informing the relevance and scope of the requirements.

Similar to the precise problem definition, the justification of the problem was based on an analysis of the results of the thematic analysis reported previously [9]. In this step, we re-examined the themes in relation to their degree of solvability (ie, whether they could realistically be addressed by digital interventions) and level of challenge (ie, the practical and organizational difficulties involved). This evaluation was informed by our knowledge of the Norwegian eHealth infrastructure and regulatory environment [19]. The purpose of this step was to assess whether the problems were significant for FACT teams and whether they represented broader structural issues in Norwegian health care.

To analyze the root causes of the problem, we used the “five whys technique” defined by Serrat [27], by starting with the basic problem and then repeatedly asking the question *why* until we reached explanations that pointed to the root causes of the problem. At each stage, the answers were informed by contextual knowledge of the Norwegian eHealth infrastructure and regulatory environment [19], ensuring that the reasoning remained realistic and grounded. This systematic approach helped distinguish surface-level symptoms from structural issues, guiding us toward a technical framing of the root problem that directly informed the design of potential solutions.

Together, the positioning, justification, and root-cause analyses established a structured understanding of the problem. These steps provided the conceptual and contextual foundations for the subsequent design work. By systematically connecting prior findings, contextual documents, and infrastructure knowledge, this phase ensured that the later definition of requirements and design of artifacts were grounded in the realities of Norwegian health care.

### Requirement Definition

Following the second step of the design science framework, we outlined an artifact that solves the explicated problem and defined requirements for the artifact. To outline the artifact, we chose the type of artifact that should be designed to solve the problem and briefly described the main functionality. To define requirements for the eHealth solutions, the first author created a table in Microsoft Excel in which each element of the problem description was entered as a separate row. For each row, the corresponding narratives from the thematic analysis were reviewed to identify the underlying user needs, workflow challenges, and contextual constraints. This table served as the analytical structure for translating empirical insights into requirements and was iteratively discussed and refined in project meetings with the coauthors.

Some requirements were derived directly from clearly stated needs within the themes, while others required further interpretation and design reasoning, where the themes pointed to broader challenges that implied more complex system functionality. For the electronic whiteboard, we additionally examined existing solutions to understand what information is typically displayed and how it supports coordination. Each requirement is linked to the corresponding element of the problem explication, ensuring transparency and traceability in how empirical insights informed the requirements. The requirements are presented at different levels of abstraction, with the electronic whiteboard receiving the most detailed specification due to its critical role in improving coordination in FACT teams. We defined functional requirements, describing what each eHealth solution can accomplish, as well as nonfunctional requirements, which apply across all the eHealth solutions.

Finally, 2 field experts with extensive experience supporting FACT teams reviewed the requirements for the electronic whiteboard. The review process involved providing written feedback on the draft requirements, followed by a meeting to clarify and discuss the comments. The experts evaluated the clarity, feasibility, and relevance of the proposed requirements. Their input was instrumental in refining the requirements, particularly by ensuring that the functionality aligned with real-world team workflows and that the terminology accurately reflected clinical practice.

### Designing Artifacts

The third step of the design science framework involves designing an artifact according to the requirements and the explicated problems [23]. In this paper, we present use cases and use case diagrams for an eHealth solution and justify the design proposals.

To generate ideas for the design of the eHealth solution, all 3 authors participated in a structured, guided brainstorming session. The session was conducted in person and facilitated by the first author, who prompted idea generation using the explicated problem, the defined requirements, and our shared knowledge of Norwegian eHealth infrastructure and regulations [19] as framing inputs.

During the session, ideas were presented, discussed, and clustered into emerging solution concepts. These concepts were then reviewed and refined based on their anticipated feasibility and usefulness in the Norwegian context (eg, alignment with existing infrastructure, compliance with regulations, and integration potential). The first author took notes throughout, capturing both the proposed ideas and the reasoning behind them.

After the session, the first author drafted the design rationale for the major design decisions, which was subsequently reviewed by the coauthors to ensure accuracy and shared agreement. Building on the selected ideas and the requirements, we developed use cases that describe the actors, goals, and interaction flows of each proposed eHealth solution. The first author also used a Unified Modeling Language (UML) 2.0 diagram [24] to visualize system functionality and relationships.

### Ethical Considerations

The project received approval from the Data Protection Official at Innlandet Hospital Trust (reference: 137877) and was reviewed by the Regional Ethical Committee, which deemed the project outside of their mandate (reference: REK Sør-Øst 104537). All participants provided signed informed consent. No patient data were gathered. The data were stored securely and deidentified. No compensation was given to the participants.

## Results

### Overview

In this section, we present the results obtained from the initial 3 steps of the design science framework. These reflect the explication of the problem to be addressed by the proposed eHealth solutions, the definition of requirements, and the creation of use cases that can guide the development of solutions.

### Explicated Problems

Problem descriptions are presented in Table 2, along with the source of each part of the description. Themes and subthemes in the table refer to the data analysis presented previously [9].

**Table 2.** Problem descriptions extracted by previously identified themes and subthemes [9].

Description part #	Source of problem description, themes, and subthemes	Problem description
1	Lack of integration	There is a lack of integration among the eHealth solutions in use by FACT^a^ teams, resulting in incomplete EHR^b^ information becoming available to the teams and impeding workflows.
2	Electronic whiteboards	Current electronic whiteboards in FACT teams are hard to use and, in some cases, lose information when transferring patients between intensive care and case management. FACT teams are not able to extract statistics from electronic whiteboards for administrative purposes.
3	EHRs	Depending on local configurations, FACT teams are not able to access EHR data from both primary and secondary care. In some cases, they use various cumbersome workarounds to access EHR data.
4	Calendars	FACT teams lack a good overview of the planned activities of their team members for administrative and safety purposes. Some calendar solutions in use are not able to track consultations that provide reimbursement.
5	Suitability of video consultations	Video consultations are not available as a tool in all FACT teams, even in situations where they are suitable for treating patients.
6	Technologies	Questionnaires used by FACT teams are paper-based, which is a hindrance to optimal workflows for storing data.
7	Documentation while traveling	FACT team members travel extensively and need to be able to access relevant eHealth solutions while outside the office.

a FACT: Flexible Assertive Community Treatment.

b EHR: electronic health record.

The precisely defined problem should also be positioned in a larger context. This context is provided in Textbox 1, which presents the purpose, stakeholders, activities, and operational environments of the teams.

Textbox 1. **Context overview of Flexible Assertive Community Treatment (FACT) teams, including stakeholders, activities, and operational environments.**

Purpose: Provide care and treatment to patients

Stakeholders: Patients, FACT team members, other health care workers, social services, and next of kin

Activities: Providing, planning, coordinating, and documenting care

Operational environments: Patients’ homes and social arenas, FACT team offices, primary care offices, and specialist care environments

In the context of this paper, we define the various stakeholders as follows:

- Patients: Persons who receive care and treatment from the FACT teams
- FACT team members: Persons who are employed in the FACT teams, and give care and treatment to patients
- Other health care workers: Persons employed in the health care service, who can provide care and treatment to patients, including general practitioners, employees in specialist health care, and employees in municipal services
- Social services: Persons employed in Norwegian social services
- Next of kin: Relatives or friends of the patient, whom the patient has authorized to assist in receiving care

The activities of the FACT teams can be divided as follows:

- Providing care: Give care to FACT team patients
- Planning care: Plan future care of patients
- Coordinating care: Coordinate the care of patients with other FACT team members and other health professionals
- Documenting care: Document care that has been given and plans in relevant systems

The operational environments are as follows:

- Patients’ homes and social arenas: Areas where patients live and live their social lives
- FACT team offices: Offices where FACT team members work
- Primary care offices: Offices for general practitioners, municipal services, and other primary care
- Specialist care environments: Hospitals and district psychiatric centers

To justify the problem, we built on our thematic analysis reported previously [9]. The analysis showed that team members of Norwegian FACT teams have highlighted the significance of the challenges associated with current eHealth solutions, by stressing the practical difficulties experienced in health care delivery, and identified the need for integrated eHealth solutions that can effectively support the complex, multidisciplinary nature of their work [9]. Although the identified challenges are technically manageable, they are complicated by the eHealth infrastructure and regulations in Norway [19]. This complexity affects not only FACT teams but also other multidisciplinary teams in Norway, indicating a general interest in improving the coordination of Norwegian health care. Together, these findings show that the problem is significant and of general interest in health care.

The results of the five whys technique are presented in Table 3. We started with the following basic problem: “FACT team members have difficulty using their eHealth solutions optimally for providing efficient care.” The technique showed that the root cause is a lack of integration between the systems in different health care sectors.

**Table 3.** Findings of the five whys technique.

Question	Answer
Why do FACT^a^ team members have difficulty using their eHealth solutions optimally for providing efficient care?	FACT team members have problems accessing some relevant data from eHealth solutions needed for providing efficient care.
Why do FACT team members have problems accessing data from relevant eHealth solutions?	FACT teams are divided into primary and specialist care, and thus have different eHealth solutions available within the team.
Why is this division a problem?	FACT team members have difficulty accessing relevant data from other health care sectors than those in which they are employed.
Why do FACT team members have difficulty accessing relevant data from other health care sectors?	Because there is a lack of communication between systems in different health care sectors.
Why is there a lack of communication between systems in different health care sectors?	Because there is a lack of integration between systems in different health care sectors.

a FACT: Flexible Assertive Community Treatment.

### Defined Requirements

In this section, we outline the artifacts that meet the explicated problem and define the functional and nonfunctional requirements for eHealth solutions for FACT teams.

**Figure 1.**
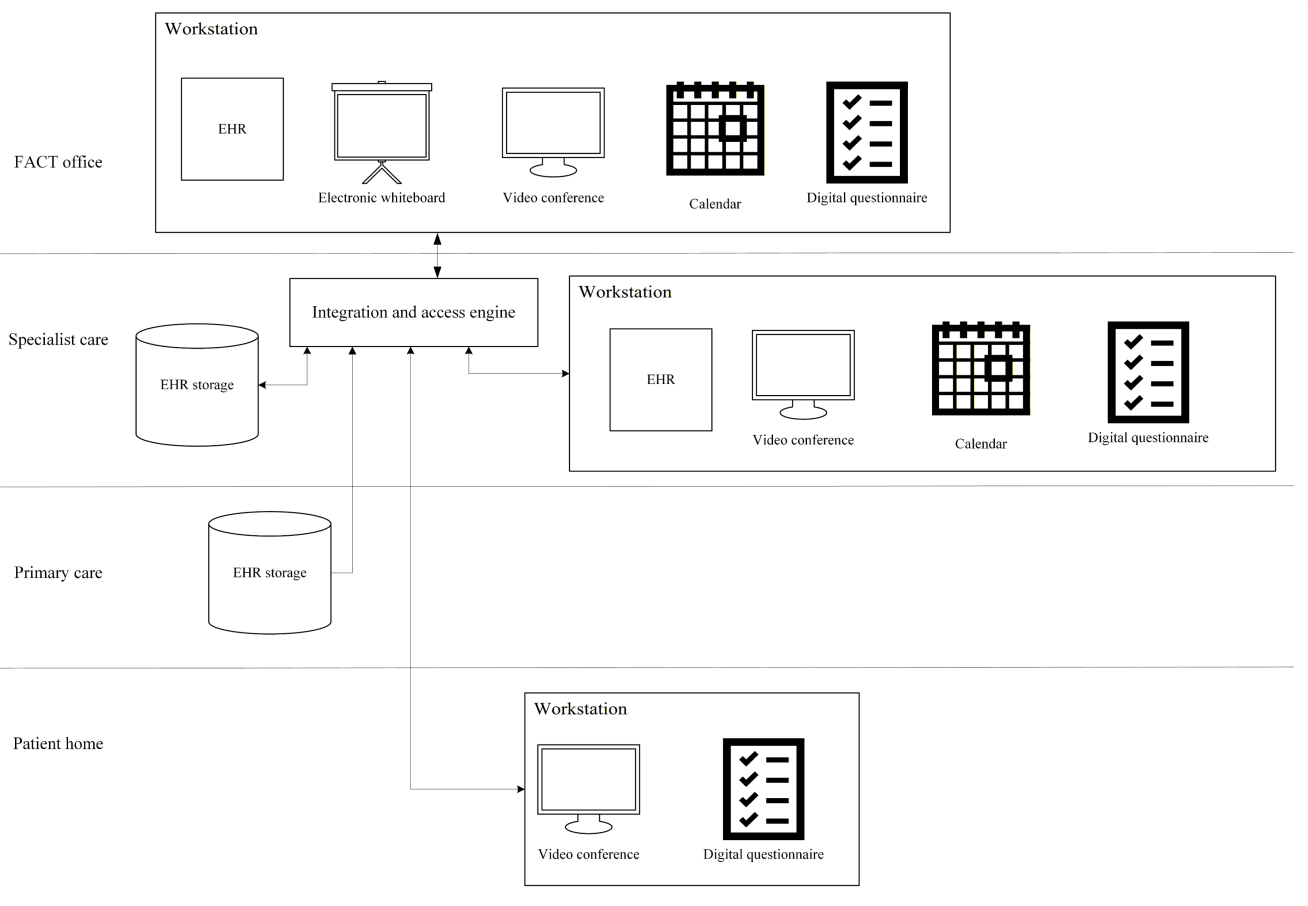
Basic outline of eHealth solutions. EHR: electronic health record; FACT: Flexible Assertive Community Treatment.

To outline the artifacts we aim to design, we classify them as instantiations (systems that can be used within a practice) [23]. These include the following eHealth solutions: EHRs, electronic whiteboards, calendars, video conferencing solutions, and digital questionnaires. EHRs represent the central storage of patient data. Electronic whiteboards are used in daily meetings to coordinate treatment and care. Calendars are used for scheduling appointments and treatments, and they provide team members with an overview of both their own calendars and the team’s calendar. Video conferencing solutions allow for meetings and video consultations, and digital questionnaires can be used to gather various patient information. To ensure that the systems are integrated, we assume that an integration and access engine is in place, which routes messages between the systems and ensures that the information is in the correct format. The basic outline and structure of these eHealth solutions are illustrated in Figure 1.

The proposed requirements and use cases are based on several underlying assumptions. Technically, the design assumes that key systems, such as EHRs and scheduling tools, can communicate through standard APIs and that local IT infrastructure allows integration of new modules. Furthermore, we assume that the necessary hardware and infrastructure are in place. Legally, it is assumed that solutions will be implemented in compliance with data protection regulations, including the General Data Protection Regulation (GDPR), and that access to patient data will follow existing legal frameworks and institutional policies. These assumptions define the boundaries of the design and should be considered when evaluating its feasibility and applicability in practice.

### Shared Functional Requirements

Certain functional requirements apply across multiple eHealth solutions, including integration with EHR systems and between calendar and video consultation solutions. These shared requirements are described here, while solution-specific requirements are detailed in the following subsections.

#### Integration With the EHR System

Electronic whiteboards, calendars, video conferencing solutions, and digital questionnaires must all be integrated with the EHR system. This ensures seamless information flow between eHealth solutions and maintains the completeness and accuracy of the patient record [28].

The source of the requirement is as follows: problem description #1 (lack of integration), #2 (electronic whiteboards), #3 (EHRs), #4 (calendars), #5 (suitability of video consultations), and #6 (technologies) (Table 2).

#### Integration Between the Video Consultation Solution and Calendar

FACT team members should be able to launch planned video consultations directly from the calendar. This enables the calendar to function as an effective tool for planning team activities.

The source of the requirement is as follows: problem description #1 (lack of integration) and #5 (suitability of video consultations) (Table 2).

### Functional Requirements: Electronic Whiteboards

#### Data Fields of the Electronic Whiteboard System

Table 4 specifies the data fields identified as required for inclusion in electronic whiteboards. These data fields include various aspects of patient information, treatment plans, and team coordination. These data fields were identified based on interviews, observations, and descriptions of existing electronic whiteboards.

**Table 4.** Data fields of an electronic whiteboard system.

Name of the data field	Description
Name	Name of the patient
Date of birth	Patient’s date of birth
Start date	Date of patient’s inclusion into the FACT^a^ team
Living situation	Description of the patient’s living situation
Phone number	Patient’s phone number
Network/family	Description of the patient’s network and family
Goals and wishes	Description of the patient’s own goals and wishes
Resources	Description of the patient’s social and practical resources
Diagnoses	Patient’s diagnoses
Legal status	Legal status related to forced treatment
Case manager	Name of the team member who is the case manager
Week plan	A plan for what contact the FACT team will have with the user for the current week
Standardized patient pathway	List of relevant standardized patient pathways allowing team members to assign the appropriate pathway to a specific patient; Overview of the pathways the patient is following, allowing tracking of progress and associated deadlines
Evaluations	Name of evaluations and when they were done
Treatment plan	Status of the treatment plan, if the patient has one, and when it was last updated
Crisis plan	Information on whether a crisis plan exists and is up to date
Why on the whiteboard?	Reason for being on the whiteboard
Questionnaires	Short description of questionnaires being used
Responsible individual	Team member responsible for the patient
Medication	Up-to-date information on the patient’s medication

a FACT: Flexible Assertive Community Treatment.

The source of the requirement is as follows: problem description #2 (electronic whiteboards) (Table 2).

#### Patient List Management

Electronic whiteboards must maintain 2 separate lists for patients receiving intensive follow-up and case management. Furthermore, team members must be able to transfer patients between these categories without any loss of information. Electronic whiteboards must allow for adding new patients and removing patients when they are no longer receiving treatment from a FACT team. This will facilitate effective care coordination, ensure comprehensive patient care, reduce the workload for team members, and minimize errors. This was identified in our observations and interviews.

The source of the requirement is as follows: problem description #2 (electronic whiteboards) (Table 2).

#### Patient Information Transfer and Elimination

Electronic whiteboards must be able to receive referral messages for the FACT teams. If a patient is accepted, relevant patient information must be directly transferred to the whiteboard from the EHR. While a patient is listed on the electronic whiteboard, team members can also add relevant patient information manually. When a patient is no longer treated by the FACT teams, their data are removed from the electronic whiteboard, and any remaining information must be transferred to the EHR. This will ensure consistency of information between EHRs and electronic whiteboards and reduce errors.

The source of the requirement is as follows: problem description #2 (electronic whiteboards) (Table 2).

### Functional Requirements: EHRs

#### Central Repository for Patient Information

A fundamental component of eHealth solutions for FACT teams is the EHR system, which must serve as the central repository for patient information, storing data from various sources. By serving as the central repository, the EHR system is able to consolidate and manage patient information from the diverse eHealth solutions used by FACT teams. This ensures a comprehensive and up-to-date view of the patient’s health data.

The source of the requirement is as follows: problem description #3 (EHRs) (Table 2).

#### Integration With eHealth Solutions and Comprehensive Data Management

The EHR system must be able to integrate with electronic whiteboards, enabling the display of relevant patient information directly within the electronic whiteboard interface. In addition to electronic whiteboards, the EHR system must be able to integrate with other eHealth solutions used by the FACT teams, such as video conferencing solutions, digital measures, and calendars [9]. This integration is a critical requirement to streamline clinical workflows.

The source of the requirements is as follows: problem description #1 (lack of integration) (Table 2).

#### Statistical Reporting

The teams should be able to extract statistical information on their work, such as the number of patients who receive intensive follow-up or case management, the number of patients with different diagnoses, and the number of patients with forced treatment. To preserve patient privacy, statistical information must be anonymized. This will allow the EHR system to be used as an administrative tool. This was identified through interviews with FACT team members.

The source of the requirement is as follows: problem description #2 (electronic whiteboards) (Table 2).

### Functional Requirements: Calendars

#### Appointment Tracking for Reimbursement

The calendar must be able to be used to track all patient consultations. This will enable calendars to be used for the tracking of treatment reimbursement.

The source of the requirement is as follows: problem description #4 (calendars) (Table 2).

#### Team Member Schedules

Team leaders should have an overview of the location of their team members. This is for both practical coordination purposes and the added safety of team members while traveling.

The source of the requirement is as follows: problem description #4 (calendars) (Table 2).

### Functional Requirements: Video Conference Solutions

#### Patient Identification

The calendar must provide identification information of the current patient to the video conference solution, to ensure that the correct patient is involved in the video consultation.

The source of the requirement is as follows: problem description #5 (suitability of video consultations) (Table 2).

#### Consultation Documentation

Calendars should create templates for documentation of consultations, which can be filled out by team members. This is because team members use calendars to keep track of their appointments with patients.

The source of the requirement is as follows: problem description #5 (suitability of video consultations) (Table 2).

#### Unplanned Video Consultation

Video conference solutions should support the ability to launch an unplanned video consultation, in addition to starting video consultations from calendars. This ensures flexibility to respond to unplanned patient needs.

The source of the requirement is as follows: problem description #5 (suitability of video consultations) (Table 2).

### Functional Requirements: Digital Questionnaires

#### Remote Patient Engagement

FACT team members should be able to send a digital questionnaire link to patients who are able to complete it independently. Alternatively, they can cooperate with patients to complete the measurement together. This allows for the distribution of digital questionnaires to patients.

The source of the requirement is as follows: problem description #6 (technologies) (Table 2).

### Nonfunctional Requirements

In addition to the functional requirements, this study identified nonfunctional requirements that define the qualities that determine how well these systems operate in practice. Unless otherwise specified, these nonfunctional requirements apply to all eHealth solutions.

#### Security and Legal Compliance

All systems must adhere to the GDPR and relevant Norwegian regulations, including the Code of Conduct for Information Security and Data Protection in Healthcare [29]. This includes secure authentication and access control. Norwegian law also grants patients the right to access their own EHRs [30]. This right also includes the data stored within electronic whiteboards, emphasizing the importance of electronic whiteboards to adhere to relevant data protection and privacy laws and regulations.

#### Multidevice Support

FACT teams are highly mobile and therefore require access to their eHealth solutions across different devices, such as PCs, tablets, and smartphones. For patient-facing tools, such as digital questionnaires and video consultations, multidevice compatibility is important to ensure participation regardless of available equipment.

#### Usability and Accessibility

Interfaces should be designed for ease of use in clinical workflows, minimizing cognitive load. Digital questionnaires and video conference solutions must accommodate patients with varying levels of digital literacy and accessibility needs.

#### Performance and Reliability

Systems must provide high levels of availability and responsive performance, particularly for video consultations. Offline functionality or synchronization mechanisms are important in settings with unstable connectivity.

#### Scalability and Interoperability

Solutions should be designed for long-term scalability and compatibility with international interoperability standards (eg, HL7 FHIR [Health Level 7 Fast Healthcare Interoperability Resources] and SNOMED CT [Systemized Nomenclature of Medicine – Clinical Terms]). This ensures future adaptability and potential use beyond Norwegian FACT teams.

### Design Rationales and Use Cases

In this section, we present the rationales behind our design choices and use cases for the proposed design.

#### Design Rationales

In Table 5, we present the design rationales of the various components of the eHealth solutions, by showing the decisions, reasons for decisions, and alternatives that were considered.

**Table 5.** Design rationales.

Part of the design	Decision	Reasons for the decision	Alternatives that were considered
Overall design	Integration between the included eHealth solutions	Data quality, accuracy, and integrity; support of modular design	—^a^
Electronic whiteboards	Patient list management	Support workflows of FACT^b^ teams	Electronic whiteboard as a built-in module of the EHR^c^
EHRs	Central repository for patient information	EHR is best suited as the central repository for patient information	—
Calendars	Team features	Supporting teamwork	Calendar as a built-in module of the EHR
Video conference solutions	Consultation documentation, unplanned video consultation, multidevice support	Support for ad-hoc consultations; support for various patient equipment	—
Digital questionnaires	Multidevice support	Support for various patient equipment	—

a Not applicable.

b FACT: Flexible Assertive Community Treatment.

c EHR: electronic health record.

#### Use Cases

**Figure 2.**
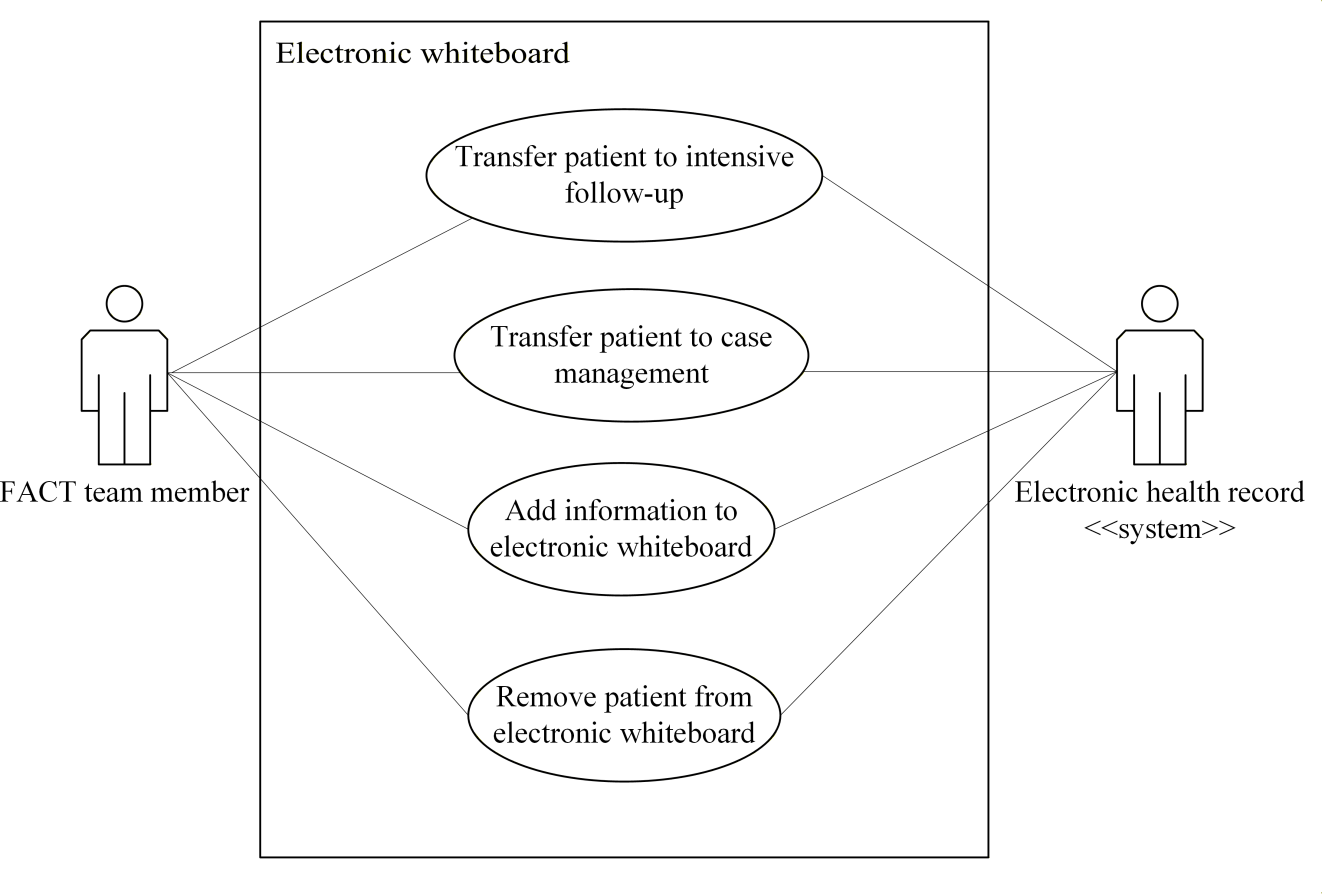
Use case for an electronic whiteboard. FACT: Flexible Assertive Community Treatment.

**Figure 3.**
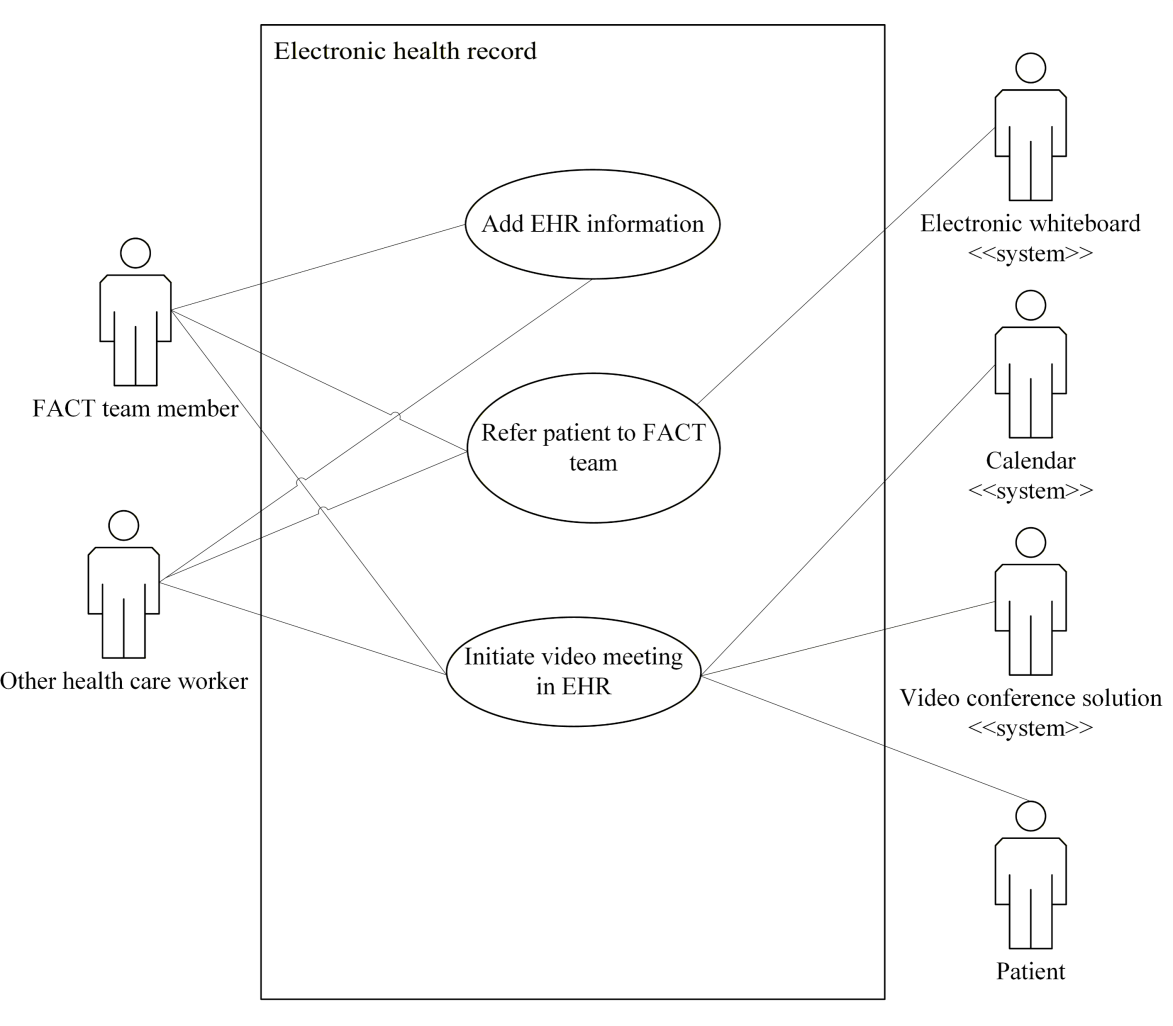
Use case for an electronic health record (EHR) system. FACT: Flexible Assertive Community Treatment.

**Figure 4.**
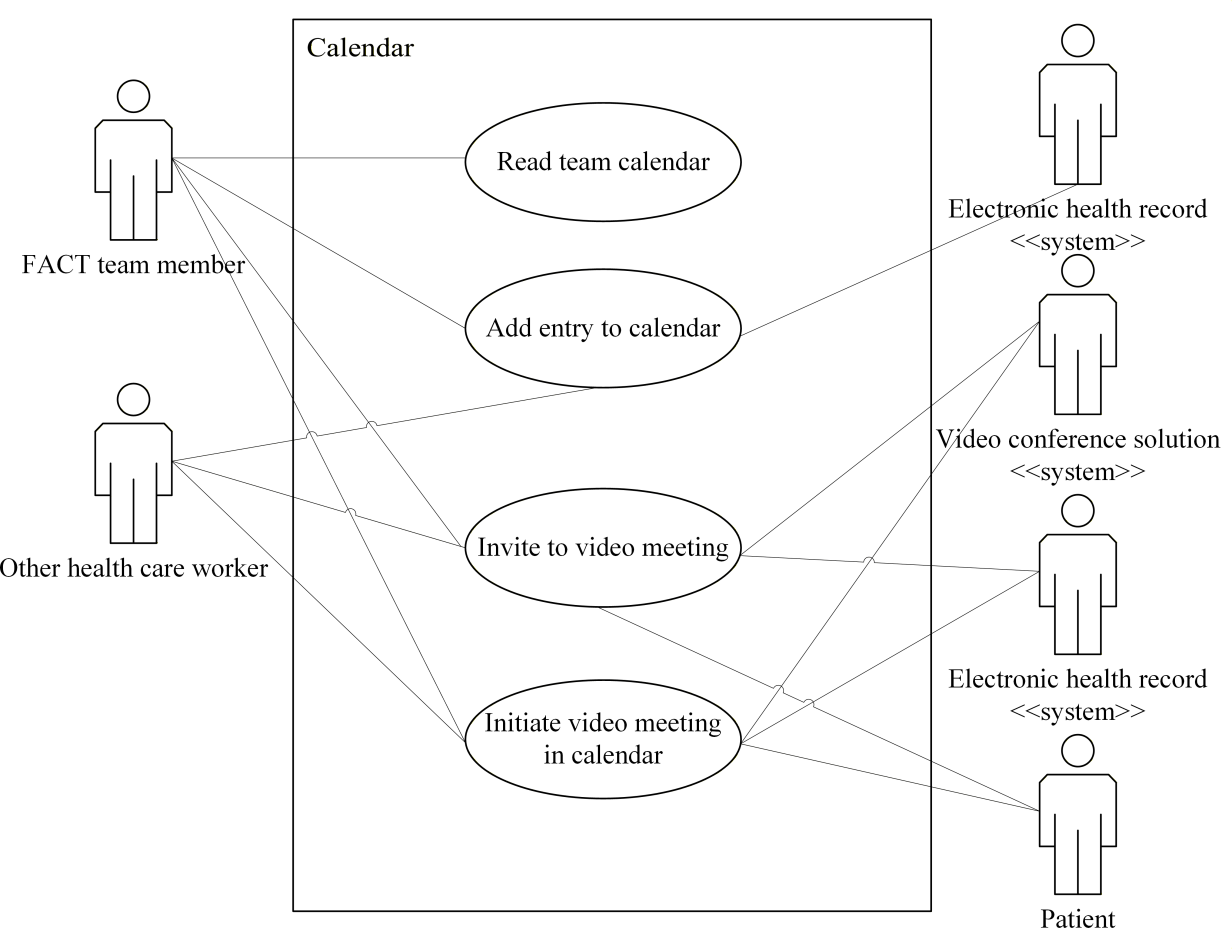
Use case for a calendar solution. FACT: Flexible Assertive Community Treatment.

**Figure 5.**
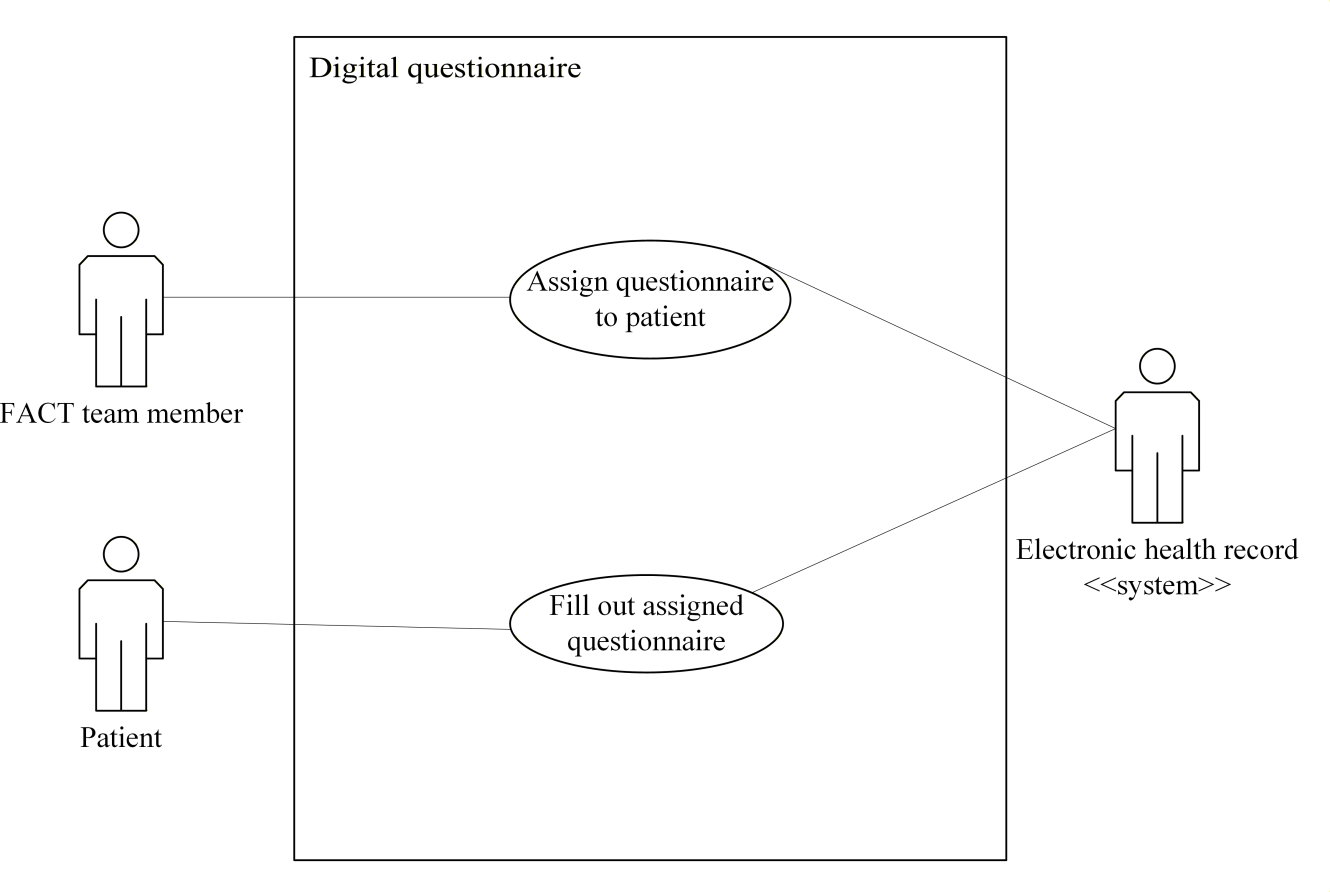
Use case for a digital questionnaire. FACT: Flexible Assertive Community Treatment.

**Figure 6.**
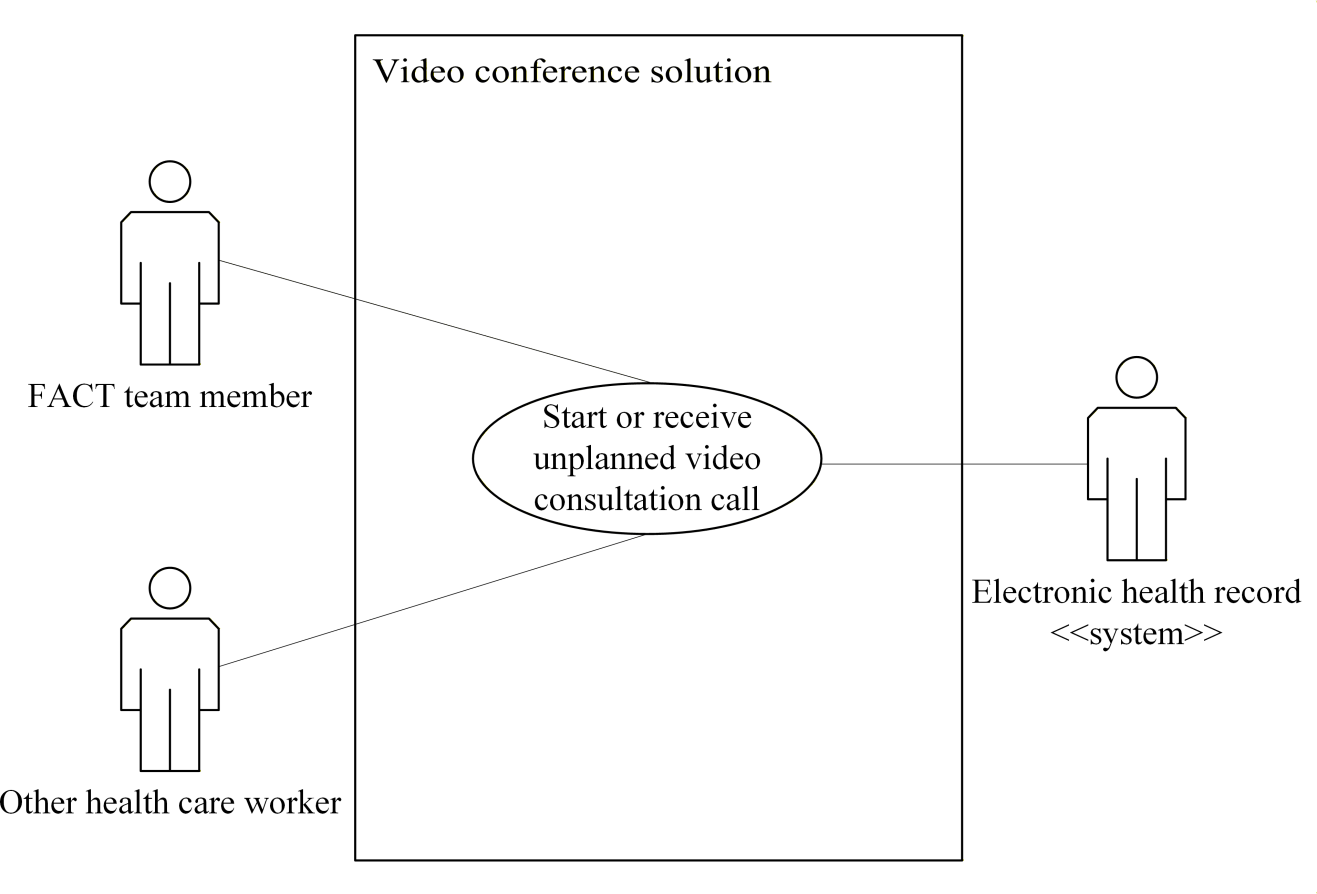
Use case for a video conference solution. FACT: Flexible Assertive Community Treatment.

Use case diagrams for the eHealth solutions are presented in Figures 2-6. These figures provide a high-level overview of the main interactions between the different systems and user groups, highlighting the functional scope of each solution. Although some use cases may involve multiple eHealth solutions, each is depicted only within the solution where it is initiated, to enhance clarity and maintain readability. Together, these figures illustrate how the individual solutions contribute to the overall integrated eHealth ecosystem.

The use case descriptions are presented below.

##### Transfer a Patient to Intensive Follow-Up

For this use case, the actor is a team member. The goal is to transfer a patient who is currently listed under individual case management on the electronic whiteboard to intensive follow-up on the whiteboard. The actions for this use case are as follows:

1. A team member selects a patient on the electronic whiteboard.
2. The team member selects an option for transferring the patient to intensive follow-up.
3. The electronic whiteboard transfers the patient from the list of patients who receive individual case management to the list of patients who receive intensive follow-up. The team member responsible for the decision is recorded. All information about the patient is retained in the system.
4. The team member commits the updated information on the electronic whiteboard to the EHR.

##### Transfer a Patient to Individual Case Management

For this use case, the actor is a team member. The goal is to transfer a patient who is currently listed under intensive follow-up on the electronic whiteboard to individual case management on the whiteboard. The actions for this use case are as follows:

1. A team member selects a patient on the electronic whiteboard.
2. The team member selects an option for transferring the patient to individual case management.
3. The electronic whiteboard transfers the patient from the list of patients who receive intensive follow-up to the list of patients who receive individual case management. The team member responsible for the decision is recorded. All information about the patient is retained in the system.
4. The team member commits the updated information on the electronic whiteboard to the EHR.

##### Add Information to the Electronic Whiteboard

For this use case, the actor is a team member. The goal is to add information about a patient to the electronic whiteboard. The actions for this use case are as follows:

1. A team member selects the electronic whiteboard entry for an identified patient.
2. The team member selects the relevant fields of the electronic whiteboard and enters new information in the fields. After completing this, they commit the information to the EHR system.
3. The information is stored on the electronic whiteboard.
4. The updated information in the electronic whiteboard is committed to the EHR.

##### Remove a Patient From the Electronic Whiteboard

For this use case, the actor is a team member. The goal is to remove a patient from the electronic whiteboard since they are no longer receiving care from the FACT team. The actions for this use case are as follows:

1. A team member selects a patient on the electronic whiteboard.
2. The team member selects an option for removing the patient from the electronic whiteboard.
3. The electronic whiteboard commits information about this decision and the identity of the team member to the EHR.

##### Add EHR Information

For this use case, the actor is a team member or other health care worker. The goal is to document the treatment of a patient in the EHR. The actions for this use case are as follows:

1. A team member selects a patient in the EHR.
2. The team member creates a new journal entry for the patient.
3. The team member adds information regarding the treatment and patient status.
4. The updated information in the EHR instance is committed to the EHR storage.

##### Refer a Patient to the FACT Team

For this use case, the actor is a health care worker. The goal is for a health care worker to refer a patient to a FACT team for possible inclusion in the team. The actions for this use case are as follows:

1. The actor writes a referral message to a FACT team, identifying themselves, the patient, and the team. Clinical information relevant to the referral is also added to the message.
2. The actor sends the referral from the EHR system to the electronic whiteboard of the FACT team.
3. FACT team members open the referral in the inbox of the electronic whiteboard.
4. The responsible FACT team members decide if the referral is accepted or rejected.
5. A FACT team member uses the electronic whiteboard to send a message back to the EHR, stating that it has been accepted.
6. The EHR sends information that is relevant for the electronic whiteboard to the electronic whiteboard. A message is sent to the referrer informing them about the decision.
7. The electronic whiteboard creates a new entry for the accepted patient. Relevant information from the EHR is added to the corresponding fields in the patient’s electronic whiteboard entry. This includes patient identification.
8. The patient is added to the list of patients who receive intensive follow-up.

An alternative flow is for rejection of the referral. If the referral is rejected, the following steps occur after step 4:

- The electronic whiteboard sends a message to the EHR about the rejection.
- The EHR sends an acknowledgment message to the electronic whiteboard, saying that the rejection has been received. The EHR then documents that the referral has been rejected and sends a message to the referrer, informing them about the decision.
- The electronic whiteboard deletes the referral after receiving the acknowledgment message.

##### Initiate a Video Meeting in the EHR

For this use case, the actor is a FACT team member or other health care worker. The goal is to start a clinical video meeting from an actor’s calendar, carry out the meeting, and document the results. Participation of a patient is optional. The actions for this use case are as follows:

1. The actor selects a patient in the EHR and selects a planned meeting consultation for the patient.
2. The actor selects an option in the EHR to start the video meeting.
3. The EHR sends a message to the video conference solution, identifying the actor and stating that they want to start or connect to the video meeting.
4. The video conferencing system starts the video meeting with the actor as a participant.
5. Other FACT team members or health care workers can also connect using a link in the calendar or in the patient’s EHR system.
6. The video conference solution creates a template for the documentation, which includes the identification of any patient and the health care workers in the conference.
7. All health care workers involved in the video meeting are granted access to this template and can fill out information during the conference.
8. After the video meeting is finished, all involved health care workers can update the video consultation documentation.
9. When all involved health care workers have approved the documentation, it is committed to the EHR by the responsible health care worker.

An alternative flow involves patient connection. If a patient is to participate in the video conference, the following steps occur after step 5:

- The video conferencing system sends a notification to the patient, saying that the video consultation has started.
- The patient clicks on the provided link to connect to the video consultation.
- The video conferencing system connects the patient to the video call.

After these steps, the flow resumes at step 6.

##### Add an Entry to the Calendar

For this use case, the actor is a team member. The goal is for the team member to add a new appointment to their calendar to keep track of appointments and activities. The actions for this use case are as follows:

1. A team member creates an entry in their calendar, detailing an appointment.
2. The entry is stored in the team member’s own calendar.
3. The entry is stored in the team calendar, including an identification of the team member.
4. Information about the appointment is transferred to the EHR and added to the patient’s record.

##### Read the Team Calendar

For this use case, the actor is a team member. The goal is for a team member to get an overview of the calendar appointments of other team members. The actions for this use case are as follows:

1. A team member accesses the team calendar section of the calendar.
2. The team member reads the calendars of the employees in the same team

##### Invite to a Video Meeting

For this use case, the actor is a FACT team member or other health care worker. The goal is to invite one or more other actors (FACT team member, patient, or other health care worker) to a planned video meeting. The actions for this use case are as follows:

1. The actor creates a calendar entry for a meeting.
2. The actor adds a time and location for the meeting to the calendar entry.
3. The calendar solution sends a message to the video conference solution, requesting a link to the video meeting.
4. The video conference solution generates a link to the video meeting and sends it to the calendar solution.
5. The calendar solution adds the link to the calendar entry for the meeting.
6. The actor sends the invitation to the other actors through the calendar solution.
7. A copy of the invitation is also sent to the EHR to store information about the upcoming video meeting there. This includes the link used to connect to the video meeting.
8. The meeting is stored in the sender’s calendar, with a link that can be used to connect to the video meeting
9. The receiver gets an invitation on their device, containing the link to the future video consultation, as an SMS text message or email. The receiver can accept or decline this invitation, in which case a message with the reply is sent back to the sender.
10. If the receiver is a FACT team member or other health care worker and accepts the invitation, the appointment is added to their calendar with the specified time for the meeting.

##### Initiate a Video Meeting in the Calendar

For this use case, the actor is a FACT team member or other health care worker. The goal is to start a clinical video meeting from the actor’s calendar, carry out the meeting, and document the results. Participation of a patient is optional. The actions for this use case are as follows:

1. The actor selects the calendar entry that contains a link to an upcoming video meeting.
2. The actor selects an option in the calendar entry to start the video consultation.
3. The calendar sends a message to the video conference solution, identifying the actor and stating that they want to start or connect to the video meeting.
4. The video conference solution starts the video meeting with the actor as a participant.
5. Other FACT team members or health care workers can also connect using a link in the calendar and the patient’s EHR system.
6. The video conference solution creates a template for the documentation, which includes the identification of any patient and the health care workers in the conference.
7. All health care workers involved in the video conference are granted access to this template and can fill out information during the conference.
8. After the video conference is finished, all involved health care workers can update the video consultation documentation.
9. When all involved health care workers have approved the documentation, it is committed to the EHR by the responsible health care worker.

An alternative flow involves patient connection. If a patient is to participate in the video conference, the following steps occur after step 5:

- The video conferencing system sends a notification to the patient, saying that the video consultation has started.
- The patient clicks on the provided link to connect to the video consultation.
- The video conferencing system connects the patient to the video call.

After these steps, the flow resumes at step 6.

##### Start or Receive an Unplanned Video Consultation Call

For this use case, the actor is a team member or other health care worker. The goal is to start an unscheduled video conference with another health care worker. The actions for this use case are as follows:

1. The actor selects a receiver for the video conference, using a unique identifier for the receiver.
2. The actor places a video call to the receiver.
3. The video conferencing solution notifies the receiver about an incoming video call and displays information about the caller.
4. The receiver gets an option to accept the video call.
5. If the receiver accepts the video call, a video conference is set up between the caller and receiver.
6. The video conference solution creates a template for the documentation, which includes the identification of any patient and the health care workers in the conference.
7. All health care workers involved in the video conference are granted access to this template and can fill out information during the conference.
8. After the video conference is finished, all involved health care workers can update the video consultation documentation.
9. When all involved health care workers have approved the documentation, it is committed to the EHR by the responsible health care worker.

##### Assign a Questionnaire to a Patient

For this use case, the actor is a team member. The goal is to assign a questionnaire for a patient to fill out. The actions for this use case are as follows:

1. A team member identifies a patient.
2. In the digital questionnaire solution, the actor selects a digital questionnaire for the patient to fill out. Information about filling out the questionnaire repeatedly is included if relevant.
3. This information is stored in the digital questionnaire solution and transferred to the EHR, which also stores the information.
4. The questionnaire solution presents the questionnaire to the patient at a relevant time.

##### Fill Out an Assigned Questionnaire

For this use case, the actor is a patient. The goal is to fill out an assigned questionnaire. The actions for this use case are as follows:

1. A patient is notified about a questionnaire that has been assigned.
2. The patient opens and completes the questionnaire. This could be with the help of team members or next of kin.
3. When the patient indicates that they are done filling out the questionnaire, the results are transferred to the EHR.
4. The EHR stores the results of the questionnaire.

## Discussion

### Summary of the Main Findings

This study aimed to identify the requirements and use cases for eHealth solutions that support teamwork in fragmented health care settings, with a focus on FACT teams. The problem analysis showed that limited access to patient data and poor integration across existing systems hinder coordinated care. From these challenges, we derived a set of requirements emphasizing interoperability and modular design. The resulting use cases illustrate how electronic whiteboards, EHRs, calendars, video conferencing solutions, and digital questionnaires can support core FACT workflows.

Although developed in a Norwegian context, the challenges of fragmented infrastructure, lack of shared information spaces, and mobile work are widely recognized across health care settings [16-18,20], indicating that the proposed requirements may be generalizable to other interdisciplinary and integrated care models.

### Discussion of the Main Results

With emerging trends like integrated care and patient-centered care, it is vital to support teamwork in health care [1,2]. In this study, the analysis showed that current digital infrastructure does not adequately support the forms of collaboration that integrated and patient-centered care rely on. The identified requirements highlight that teamwork requires systems that enable continuous coordination across distributed actors and settings. These findings situate the need for technical integration within a broader, workflow-oriented perspective, in which the goal is to connect systems and to support the collaborative practices that define integrated and patient-centered care.

Efforts to address integration challenges in health care more broadly provide useful context for our work. Kinast et al [21] have defined a comprehensive set of requirements for data integration, encompassing data acquisition, processing, analysis, storage, and security. Their work focuses on the technical and data management layers of integration, thereby ensuring that information can be exchanged reliably and securely across systems. In contrast, this study addresses a higher level of abstraction, defining requirements and use cases that support team-level coordination and shared clinical workflows. The Valkyrie project offers another perspective by proposing a prototype ICT architecture centered on a virtual health record that provides cross-level access to patient data [22]. Such an infrastructure could enable the implementation of many of the requirements identified in this study, particularly those related to shared information spaces and cross-organizational coordination.

Our findings suggest that effective support for teamwork in FACT settings depends on an eHealth architecture that enables reliable interoperability across the diverse systems used in daily workflows. The proposed integration and access engine addresses this need by supporting both syntactic and semantic interoperability through established standards such as HL7 FHIR, SNOMED CT, and ICD (International Classification of Diseases). Because primary and specialist care in Norway rely on heterogeneous IT platforms, the requirements were formulated at a system-independent level to support interoperability across sector-specific infrastructure.

Key design decisions and their rationales are summarized in Table 4. Rather than embedding all functionality within the EHR, we chose a modular approach in which tools, such as electronic whiteboards and calendars, operate as separate systems but integrate through shared standards. This reflects the need for flexible, task-specific views that support daily coordination in FACT teams while still ensuring that essential information remains available within the core clinical record. Some functional overlap remains, for example, planned consultations appear in both the calendar and the EHR. However, this redundancy helps maintain continuity of documentation. While consistency between systems is critical, detailed mechanisms for synchronization, conflict resolution, and data ownership were beyond the scope of this study and would need to be addressed during subsequent technical design and implementation. Similarly, enabling video consultations to be initiated from either system supports the varied work practices of teams and preserves the EHR as a central workspace. Calendars retain integration with video conferencing systems to facilitate scheduling without constraining the modular architecture.

Beyond integration and system design, several of the identified requirements focus on the workflows of FACT teams, such as managing patient lists, conducting video consultations, and assigning digital questionnaires. These ensure that essential functionalities are in place before addressing nonfunctional requirements, such as accessibility across devices and patient inclusion.

A nonfunctional requirement is the availability of eHealth solutions on a range of devices. This is required since FACT team members often travel to meet their patients and therefore need access to eHealth solutions on mobile devices. For eHealth solutions that involve patients, it is important that the patients can access the solutions on their devices. While this study focused on the perspectives of health care professionals, the requirements for digital questionnaires and video consultations involve patient interaction. In practice, not all patients are able or willing to complete questionnaires independently, which highlights the need for flexible solutions that allow for assisted reporting when necessary. Similarly, the suitability of video consultations in psychiatric care varies across patients and situations, as discussed in previous research [9]. Their use in psychiatric care therefore requires clinical judgment, as limitations in assessing nonverbal cues and managing acute situations may pose safety risks.

Building on these requirements, we defined use cases for the eHealth solutions and illustrated the use cases in UML use case diagrams. These use cases were helpful in detailing the functionality of each solution and can serve as a foundation for communication with stakeholders and for guiding future development efforts.

### Strengths and Limitations

A key strength of this study is its grounding in real-world clinical practice. The design science approach enabled us to build on empirical insights from prior observations and interviews of FACT teams, complemented by document analysis and contextual knowledge of the Norwegian eHealth infrastructure. By structuring the work through the iterative steps of problem explication, requirement definition, and design of use cases, the study adhered to core design science principles that emphasize problem-driven development closely linked to practice. This ensured that the proposed eHealth solutions were informed by the actual workflows, constraints, and coordination challenges experienced by FACT teams.

While the study provides a structured foundation for designing eHealth solutions for FACT teams, several limitations must be acknowledged. First, the requirements and use cases were derived through document analysis, thematic analysis of prior empirical material, and expert review, but were not validated through direct empirical testing with end users. This limits certainty about how well the proposed solutions align with real-world practices. Although grounding the work in existing evidence and expert input helped mitigate this limitation, future studies should include workshops, usability testing, and pilot implementations. Second, the design recommendations were developed within the context of the Norwegian eHealth infrastructure. As a result, adaptations may be necessary in countries with different infrastructure. However, challenges, such as fragmented systems and limited data access, are widely reported, suggesting that the underlying design principles may still be transferable. Third, the study focused primarily on the perspectives and needs of health care professionals. While patient-facing functionalities, like digital questionnaires and video consultations, were considered, direct patient involvement fell outside the present scope. This may have constrained the identification of needs related to usability, digital literacy, and patient experiences. Future work should therefore incorporate input from patients and caregivers. Fourth, the study did not consider financial or policy-level feasibility. Evaluating costs, sustainability, and prioritization will be important in future work to inform decisions about implementation at scale.

### Conclusion

This study shows how current eHealth solutions influence teamwork and care coordination in FACT teams and how digital support can reduce fragmentation and improve information flow. The requirements and use cases developed here provide a foundation for designing solutions that align with real-world workflows and the needs of multidisciplinary teams.

Although grounded in the Norwegian context, the core design principles of interoperability, timely access to information, and support for distributed decision-making are relevant across health care settings. The findings therefore have implications for not only technical development but also policy, highlighting the importance of digital infrastructure that actively enables collaborative work.

More broadly, the study illustrates how workflow-oriented design can strengthen integrated and patient-centered care models that rely on effective teamwork. By focusing on how technology can support collaboration, the work offers insights applicable to multidisciplinary health care teams beyond FACT.

## Abbreviations

API: application programming interface
EHR: electronic health record
FACT: Flexible Assertive Community Treatment
GDPR: General Data Protection Regulation
HL7 FHIR: Health Level 7 Fast Healthcare Interoperability Resources
ICD: International Classification of Diseases
ICT: information and communications technology
SNOMED CT: Systemized Nomenclature of Medicine – Clinical Terms
UML: Unified Modeling Language
